# Exploration of the role of the virulence factor ElrA during *Enterococcus faecalis* cell infection

**DOI:** 10.1038/s41598-018-20206-6

**Published:** 2018-01-29

**Authors:** Natalia Nunez, Aurélie Derré-Bobillot, Stéphane Gaubert, Jean-Marie Herry, Julien Deschamps, Yu Wei, Thomas Baranek, Mustapha Si-Tahar, Romain Briandet, Pascale Serror, Cristel Archambaud

**Affiliations:** 1 Micalis Institute, INRA, AgroParisTech, Université Paris-Saclay, 78350 Jouy en Josas, France; 2 Département de Virologie, Institut Pasteur, 28 rue du Dr. Roux, 75015 Paris, France; 3 INSERM, Centre d’Etude des Pathologies Respiratoires (CEPR), UMR 1100 Tours, France; 4 Université François Rabelais, Tours, France; 5 Present Address: Unit of Hepatitis B Virus and Liver Disease, Institut Pasteur of Shanghai, Chinese Academy of Sciences, 320 Yueyang Road, 200031 Shanghai, China

## Abstract

*Enterococcus faecalis*, an organism generally not pathogenic for healthy humans, has the potential to cause disease in susceptible hosts. While it seems to be equipped to interact with and circumvent host immune defense, most of the molecular and cellular mechanisms underlying the enterococcal infectious process remain elusive. Here, we investigated the role of the Enterococcal Leucine Rich protein A (ElrA), an internalin-like protein of *E. faecalis* also known as a virulence factor. ElrA was previously shown to prevent adhesion to macrophages. We show that ElrA does not inhibit the basic phagocytic process, but is able to prevent sensing and migration of macrophages toward *E. faecalis*. Presence or absence of FHL2, a eukaryotic partner of ElrA, does not affect the ElrA-dependent mechanism preventing macrophage migration. However, we highlight a partial contribution of FHL2 in ElrA-mediated virulence *in vivo*. Our results indicate that ElrA plays at least a dual role of which anti-phagocytic activity may contribute to dissemination of extracellular *E. faecalis* during infection.

## Introduction

Gram positive *Enterococcus faecalis* is a subdominant commensal of the gut microbiota. Following antibiotic-mediated alteration of the intestinal microbiota, also known as dysbiosis, *E. faecalis* outgrows and becomes a dominant pathobiont^1^. Taking advantage of the host altered immune system, it translocates through the gut barrier and disseminates to the bloodstream and to deeper organs^2^. Nowadays, enterococci rank as the third most-important cause of nosocomial infections, in which *E. faecalis* is responsible for more than 60% of hospital-acquired enterococcal infections^3^. The pathogenic potential of *E. faecalis* relies on multifactorial mechanisms that include the response to an altered environment, the outgrowth and the switch from a symbiontic to a pathogenic lifestyle promoted by the expression of virulence factors^2,4^.

The enterococcal leucine-rich protein A (ElrA), which belongs to the internalin family, is a surface virulence factor^5^. Internalins were first identified in *Listeria monocytogenes* and are well-defined virulence factors^6^. They are characterized by the presence of leucine-rich repeats (LRRs). ElrA is an 80 kDa protein with an export signal peptide, ten LRRs and a C-terminal WxL domain for non-covalent binding to peptidoglycan^7^. ElrA is encoded as the first gene of a five-gene operon (*elrA-E* operon) activated by the positive regulator ElrR^8^. The other four genes of the operon (*elrB*, *elrC*, *elrD* and *elrE*) encode four proteins with an LPXTG domain, two WxL domains and a transmembrane domain, respectively. Considering the cell wall binding domains present, this operon is predicted to encode a protein complex at the bacterial surface^9^. The current knowledge is that genes *elrB*, *elrC*, *elrD* and *elrE* are required for ElrA stabilization^9^. Importantly, expression of the *elrA* gene is not detected *in vitro* but is induced *in vivo*^5^. Disruption of *elrA* significantly attenuates bacterial virulence in a peritonitis model of infection in ICR mice as well as in Balb/C mice infected by the intraperitoneal, intravenous, and transurethral routes^5,9,10^. ElrA has also been shown to modify host inflammatory responses by increasing IL-6 release *in vivo*^5,8^. Since *elrA* expression is undetectable *in vitro* and *elrB*, *elrC*, *elrD* and *elrE* are required for ElrA stabilization, the native promoter of the operon has been exchanged by the constitutive *aphA3* promoter (P^+^-*elrA-E* strain)^9^. The corresponding strain expresses the *elrA-E* operon constitutively and is more virulent^9^. In addition, ElrA prevents adhesion to macrophages^9^. Recently, a physical interaction between ElrA and FHL2, a human member of the four-and-a-half-LIM-only protein family has been revealed using a two-hybrid screen in yeast^10^. Interestingly, deletion of the FHL2-interacting domain of ElrA decreased *E. faecalis* infectivity in Balb/c mice after intravenous and urinary tract infections^10^. FHL2 is formed of LIM domains involved in several protein-protein interactions and participates in a wide range of cellular processes including cellular transcription, signal transduction and cell survival or death^11^. Such broad roles for FHL2 opens up a wide spectrum of possibilities for FH2 contribution in *E. faecalis* virulence. Moreover, while the role of ElrA in *E. faecalis* virulence is clearly established, how ElrA-expressing *E. faecalis* escapes macrophage phagocytosis remains poorly understood.

In this study, we aimed to further investigate ElrA-dependent mechanisms that prevent *E. faecalis* binding to macrophages and favor *E. faecalis* evasion from macrophage phagocytosis. Using the genetically modified P^+^-*elrA-E* strain and isogenic strains expressing or not expressing ElrA, we assessed the capacity of other cell types to internalize ElrA-expressing *E. faecalis*. We also evaluated whether ElrA modifies the structure of the enterococcal population or bacterial surface properties in such a way that macrophages would not be able to recognize *E. faecalis*. We investigated whether disruption of FHL2, an ElrA partner, impacts on this process.

## Results

### ElrA expression impacts upon structure of the enterococcal population without major changes in *E. faecalis*

The *elrA-E* operon is predicted to encode a protein complex at the bacterial surface, where ElrA expression may increase surface negative charge during cell infection to prevent macrophage adhesion^5,9^. Here, we performed a series of *in vitro* assays to establish which observable characteristics conferred by ElrA expression may be prevailing to prevent cell adhesion and ultimately macrophage phagocytosis.

First, to assess if ElrA expression modifies *E. faecalis* surface charge, we evaluated the electric potential at the bacterial surface, known as zeta potential. Experiments were carried out on four isogenic strains expressing or not expressing ElrA (Table 1 and Supp. Figure 1). Bacterial surface components are ionized according to environmental pH. The use of an electric field triggers bacterial migration towards the electrode of opposite charge of its global charge with a velocity proportional to the magnitude of the zeta potential^12^. We thus measured the electrophoretic motility of these four *elrA* isogenic strains, at pH 7, like in conditions of cell infection. Using the Smoluchowski equation^13^, we determined the zeta potential values (ζ) as follows ζ(OG1RF) = −36.2 ± 1.0 mV, ζ(P^+^-*elrA-E*) = −29.9 ± 2.8 mV, ζ(P^+^-Δ*elrA*) = −34.8 ± 1.4 mV, ζ(CPL-*elrA*) = −32.9 ± 2.2 mV. All the tested strains have a dominant negative charge consistent with an electronegative zeta potential value, indicating that in conditions of cell infection, where the pH is neutral, ElrA expression does not increase surface negative charge.

**Table 1:** ElrA isogenic strains used in this work.

Strains	Description	Origin
OG1RF	Plasmid free wild-type strain No *elrA-E* expression	^39^
P^+^-*elrA-E*	OG1RF P_*aphA3*_::*elrA-E* *elrA-E* expression	^9^
P^+^-Δ*elrA*	OG1RF P_*aphA3*_::Δ*elrA* Only *elrB-E* expression	^9^
CPL-*elrA*	OG1RF P_*aphA3*_::Δ*elrA* + pVE14047 (P_*usp45*_::*elrA*) Complementation of P^+^-Δ*elrA*	This work

The OG1RF wild-type strain does not express the *elrA-E* operon *in vitro*; the P^+^-*elrA-E* strain constitutively expresses the whole operon; the P^+^-Δ*elrA* strain constitutively expresses *elrB-E*, but not *elrA*; and the CPL-*elrA* strain corresponds to P^+^-Δ*elrA* complemented for *elrA* expression.

**Figure 1:**
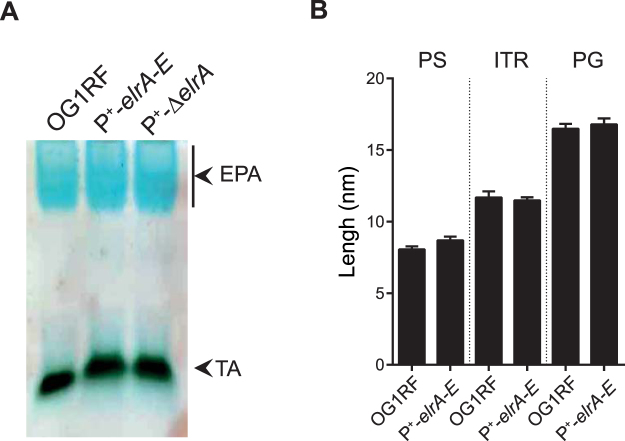
Effect of ElrA expression on *E. faecalis* envelope (**A**) Cell wall polysaccharide profiles from the OG1RF, P^+^-*elrA-E* and P^+^-Δ*elrA* strains recovered in exponential growth phase. Abbreviations: EPA, Enterococcal Polysaccharide Antigen; TA, teichoic acids. (**B**) Thickness of the polysaccharide pellicle (PS), the intermediate translucent region (ITR) and of the peptidoglycan layer (PG) was measured from electronic microscopy images using Image J software. Three measurements were made per bacteria. Mean values and SEM were calculated on 10 independent bacteria. Statistical analysis was performed using unpaired Student’s t test.

We also investigated whether unlocking expression of the *elrA-E* operon induces a modification of the cell wall composition. Both P^+^-*elrA-E* and P^+^-Δ*elrA* strains exhibit a similar profile to that of the wild type strain with a cyan blue band corresponding to the rhamnopolysaccharide Epa and a black band corresponding to the teichoïc acids (Fig. 1A). These results showed that the major composition of surface polysaccharides is conserved in the *elrA* isogenic strains. Moreover, measurement of the different layers of the bacterial envelope by transmission electron microscopy showed that both wild type and P^+^-*elrA-E* strains had similar thickness for the polysaccharide pellicle (8.1 ± 0.8 nm and 8.7 ± 1.0 nm, respectively), the intermediate translucent region (11.7 ± 1.6 nm and 11.5 ± 0.8 nm, respectively) and the peptidoglycan layer (16.5 ± 1.3 nm and 16.8 ± 1.6 nm, respectively) (Fig. 1B). The morphology and structure of the cell wall as well as the content of surface polysaccharides between strains thus remain identical whether or not expressing ElrA.

**Figure 2:**
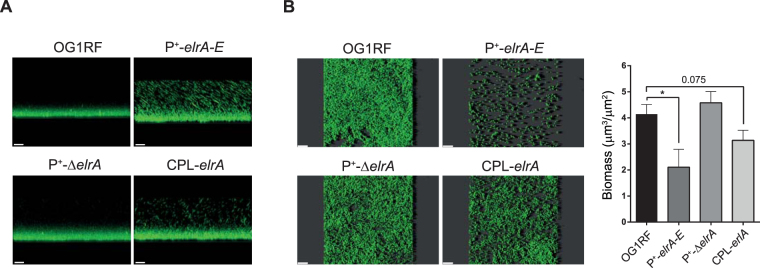
Effect of ElrA expression on *E. faecalis* biofilm (**A**) Biofilms of the OG1RF wild-type strain, the P^+^-*elrA-E* strain, the P^+^-Δ*elrA* and the CPL-*elrA* strain were observed after 6 h incubation and growth at 37 °C without washes. (**B**) Right panel: Biofilms of ElrA isogenic strains were observed 6 h post-inoculation in BHI and after washes. Left panel: Biomass was calculated from the raw images using Image J software and Comstat plugin. Data are represented as mean with SEM from two independent experiments. Statistical analysis was performed using unpaired Student’s t test. Asterisks indicate a p-value considered statistically significant (*P < 0.05). All biofilms were observed by confocal microscopy; white scale bars, 20 μm. Image projections were obtained using IMARIS software.

Next, we compared the development and the cohesion of biofilms formed by *E. faecalis* grown statically using confocal microscopy. We first examined the biofilms formed by the different strains in the absence of washes. While very few floating bacteria were found with the OG1RF and P^+^-Δ*elrA* strains, the P^+^-*elrA-E* and CPL-*elrA* strains remained mainly planktonic and floating bacteria were observed, suggesting that ElrA-expressing *E. faecalis* does not form a solid biofilm (Fig. 2A). The strength of the different biofilms was followed after bacterial incubation and washes. We observed that biofilms of the OG1RF and P^+^-Δ*elrA* strains were formed and well-attached to the well surface (Fig. 2B, left panels). In contrast, biofilms of the P^+^-*elrA-E* strain was easily disrupted after the washes and bacteria were poorly attached to the abiotic surface. In agreement with these observations, the corresponding biomass of the P^+^-*elrA-E* strain was ~50% thinner compared to the wild type and P^+^-Δ*elrA* strains (Fig. 2B, right panel). A similar trend was observed with the CPL-*elrA* strain (Fig. 2B, lower rightest panel). Consistently, the P^+^-*elrA-E* strain produced biofilms were 3 times less cohesive than those of the wild type and P^+^-Δ*elrA* strains (Supp. Figure 2), indicating that ElrA promotes a loss of cohesion between bacteria.

No notable differences regarding charge, morphology, cell wall structure and surface polysaccharides content was noted between strains, indicating that ElrA expression does not induce major changes that interfere with macrophage recognition. However, ElrA promotes a loss of cohesion between bacteria triggering a free-floating state that may affect contact with macrophages.

### ElrA confers an anti-phagocytic activity when bacterial contact with macrophages and non-professional phagocytic cells is forced

**Figure 3:**
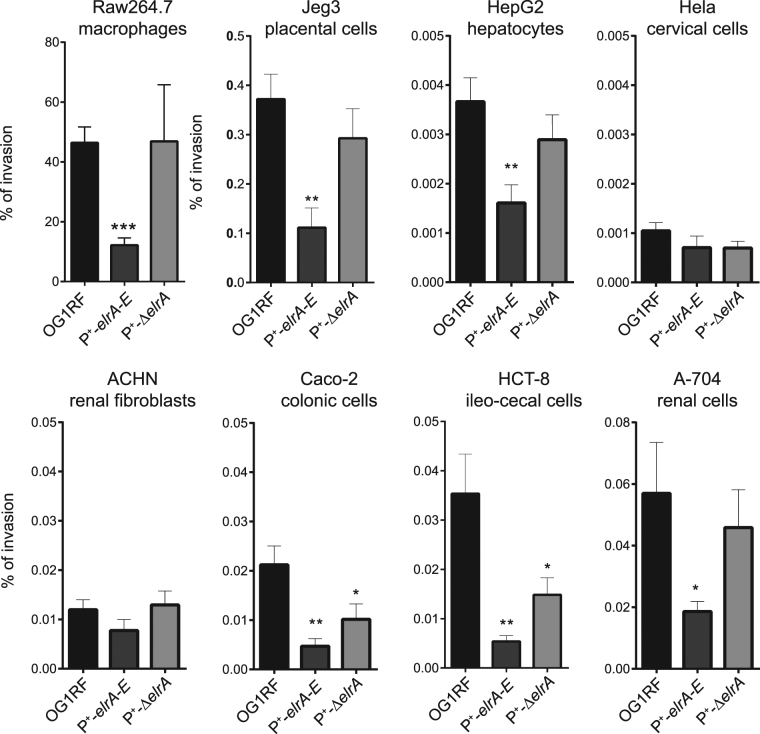
Effect of ElrA-expressing *E. faecalis* on host cells. Exponentially growing *E. faecalis* strains were used to infect Raw264.7 macrophages and placental cells (Jeg-3), hepatic cells (HepG2), epithelial cervical (Hela) cells, fibroblastic kidney cells (ACHN), colonic epithelial cells (Caco-2), ileocecal epithelial cells (HCT-8), and epithelial kidney cells (A-704). Percentage of invasion was determined as the ratio of intracellular bacteria to the initial inoculum. Data are represented as mean with SEM of at least 3 independent experiments for each cell line. Statistical analysis was performed using unpaired Student’s t test. Asterisks indicate a p-value considered statistically significant (*P < 0.05; **P < 0.01; ***P < 0.001).

To test whether forcing contact of bacteria to macrophages would circumvent the lack of adherence^14^, we decided to constrain bacteria to reach macrophages in a phagocytosis assay where bacteria were centrifuged with the cells. The capacity of ElrA-expressing *E. faecalis* to be internalized into macrophages was evaluated by determining the percentage of invasion as the ratio of internalized bacteria 1 hour post-infection (p.i.) relative to the initial inoculum. Professional phagocytic Raw264.7 macrophages engulfed 50% of the initial inocula of the wild type and P^+^-Δ*elrA* bacteria (Fig. 3). Strikingly, less than 20% of the P^+^-*elrA-E* bacteria were internalized, showing that, although forcing contact, the efficiency of P^+^-*elrA-E* bacteria adhesion or internalization was lower than that of the wild type. These data indicated that ElrA still confers an anti-phagocytic activity even when bacteria are in contact with macrophages.

During infection, *E. faecalis* also encounters non-professional phagocytes. We investigated whether ElrA-mediated escape was a more general mechanism. We performed the same invasion assay in seven human cell lines, all derived from tissues potentially targeted by *E. faecalis* during host infection. In contrast to what we observed for macrophages, the percentage of internalized wild-type *E. faecalis* was 2-4 orders of magnitude lower in non-immune cells (Fig. 3). The percentage of invasion reached almost 0.4% in placental cells (Jeg-3), but was only about <0.004% in hepatic cells (HepG2) and ~0.001% in epithelial cervical (Hela) cells. It oscillated between 0.01% and 0.05% in fibroblastic kidney cells (ACHN), in colonic epithelial cells (Caco-2), in ileocecal epithelial cells (HCT-8) and in epithelial kidney cells (A-704). Except for cervical epithelial and renal fibroblastic cells, we recovered a significant lower number of internalized P^+^-*elrA-E* bacteria compared to the wild type strain and to the P^+^-Δ*elrA* strain (Fig. 3). These results showed that ElrA expression prevents *E. faecalis* internalization in various cell types, suggesting that ElrA-mediated escape is a general mechanism to circumvent host defense.

### ElrA does not block phagocytosis per se, but impairs migration of macrophages toward ElrA-expressing *E. faecalis*

**Figure 4:**
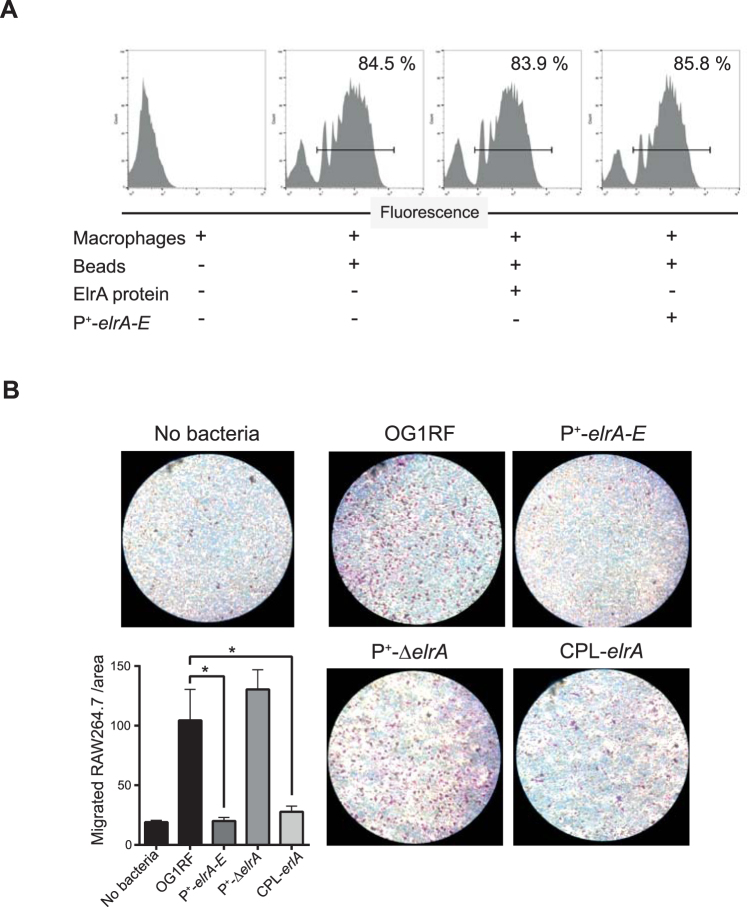
Effect of ElrA on macrophage phagocytosis and migration (**A**) Raw 264.7 macrophages were incubated with fluorescent beads and incubated with P^+^-*elrA-E* or 10 nM of purified ElrA protein for 3 h prior to incubation with fluorescent beads. Histograms represent the distribution of macrophages phagocytizing fluorescent beads. Percentage of bead-positive macrophages was calculated by gating on live macrophages (not shown). Representative flow cytometry histograms from two independent experiments are shown. (**B**) Cell migration was observed after ~20 h of incubation on an inverted light microscope. Macrophages trapped in the membrane are stained with Giemsa coloration. Representative field of three independent experiments are shown. Migrating Raw264.7 macrophages were counted in five random fields per well at 20× magnification. Data are represented as mean with SEM of three independent experiments. Asterisks indicate a p-value considered statistically significant (*P < 0.05).

Because forced contact is not sufficient to restore full adhesion of ElrA-expressing *E. faecalis* to host cells, we tested whether ElrA hampers macrophage capacity to engulf particles. We incubated macrophages with the P^+^-*elrA-E* strain, which constitutively expresses the operon, or with purified ElrA prior to incubation with fluorescent beads and quantified the percentage of bead-positive macrophages by flow cytometry. We found that around 85% of untreated macrophages were able to phagocytize fluorescent beads (Fig. 4A). When incubated with the P^+^-*elrA-E* strain or purified ElrA, macrophages phagocytized beads with the same efficacy, demonstrating that neither released nor surface-bound form of ElrA interfered with macrophage capacity to phagocytize. These results showed that ElrA does not inhibit the intrinsic capacity of macrophages to phagocytize, suggesting that ElrA may hinder mechanisms upstream of the phagocytic process, such as bacterial sensing by macrophages.

To test this hypothesis, we used a modified Boyden chamber, such that macrophages were seeded in the upper compartment of the chamber and one of the four *E. faecalis* isogenic strains expressing or not ElrA were incubated in the lower chamber. In this assay, macrophages are entrapped in the membrane separating the two compartments if they sense and migrate toward the bacteria in the lower chamber. The results showed that 104 ± 26 macrophages migrated toward bacteria when exposed to the *E. faecalis* wild type strain; this rate of migration was 5-fold higher than random migration of macrophages in the absence of infection (P = 0.0112) (Fig. 4B). Similar numbers of migrating OG1RF-exposed macrophages were counted when exposed to the P^+^-Δ*elrA* strain. Strikingly, we observed that macrophages did not migrate toward the P^+^-*elrA-E* and CPL-*elrA* strains, both overexpressing ElrA. In a similar way to what we observed in the absence of infection, only 20 ± 5 macrophages migrated in these conditions (Fig. 4B). This finding showed that macrophages do not migrate toward ElrA-expressing *E. faecalis*, indicating that ElrA-expressing *E. faecalis* are not sensed by macrophages.

### Inhibition of macrophage migration is independent of FHL2-ElrA interaction

**Figure 5:**
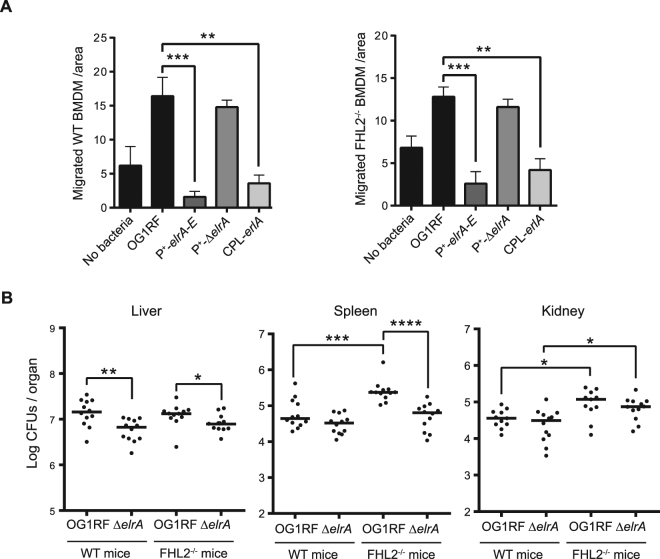
Effect of FHL2-ElrA interaction in *E. faecalis* infection. (**A**) Migrating macrophages (Left panel, wild type bone-morrow derived macrophages or BMDMs; right panel, FHL2^−/−^ BMDMs) were counted in five random fields per well at 20 × magnification. Data are represented as mean with SEM. Statistical analysis was performed using unpaired Student’s t test on a representative experiment from two independent experiments. (**B**) C57BL/6 wild type and FHL2^−/−^ mice were infected intravenously with 5 × 10^8^ CFUs of OG1RF wild type or Δ*elrA* strain. The liver, the spleen, and the kidneys were harvested 24 hours post-infection and serial dilutions of organ homogenates were plated on selective enterococci agar medium (BEA). Each dot corresponds to one mouse (n = 12) and were obtained from three independent experiments. Black bars represent median values. Statistical analysis was performed using Mann-Whitney test on three independent experiments. Asterisks indicate a p-value considered statistically significant (*P < 0.05, **P < 0.01, ***P < 0.001).

ElrA can be both exposed at the bacterial surface and released in the culture supernatant^7^. A eukaryotic interactive target of ElrA is FHL2^10^. We investigated whether FHL2-ElrA interaction was involved in preventing macrophage migration. The migration assay was performed with bone-marrow macrophages (BMDMs) differentiated from C57BL/6 J wild type or FHL2^−/−^ mice. In both cases, we observed that BMDMs did not migrate toward ElrA-expressing *E. faecalis* (Fig. 5A). These data substantiate the effect of ElrA in inhibiting sensing and migration of macrophages, albeit independently of FHL2.

Because deletion of the FHL2-interacting domain of ElrA impacts infection *in vivo*^10^, we investigated the role of FHL2 during *E. faecalis* infection by challenging FHL2^−/−^ mice versus wild-type C57BL/6 mice upon intravenous infection for 24 h with *E. faecalis*. Both mice were challenged with OG1RF and the Δ*elrA* strain since ElrA is expressed *in vivo*^5^. Different trends were observed depending on the organ invaded by *E. faecalis* (Fig. 5B). FHL2 was found to play a role in defense against *E. faecalis* in the spleen and in the kidneys but not in the liver. In the spleen, FHL2 defense against *E. faecalis* would be triggered by ElrA. Together, these data showed that FHL2 is involved in controlling *E. faecalis* infection and FHL2 may counteract ElrA action in the spleen to prevent *E. faecalis* burden. The different phenotypes observed *in vivo* among the various organs suggest that ElrA and FHL2 have differential roles depending on the conditions encountered by *E. faecalis* during infection.

## Discussion

ElrA is an internalin-like protein implicated in prevention of macrophage adhesion and *E. faecalis* evasion from macrophage phagocytosis^9^. In this study, we explored the mechanism of action of ElrA. We ruled out the possibilities that electric repulsion between the bacterial and eukaryotic cell surface or that major structural changes in *E. faecalis* envelope are responsible for ElrA-dependent escape. ElrA confers a free floating state to ElrA-expressing bacteria, which is not responsible for the inability of *E. faecalis* to adhere to macrophages. Moreover, we showed that ElrA does not block macrophage capacity to phagocytize. Instead, we demonstrated that macrophages remain insensitive to and do not migrate towards ElrA-expressing *E. faecalis*. Of note, ElrA-mediated escape occurs in some non-professional phagocytic cells. We also investigated the role of FHL2, a eukaryotic partner of ElrA. *In vivo*, we showed that FHL2 is involved in *E. faecalis* infection, but with a partial contribution relying on its interaction with ElrA, suggesting that FHL2-ElrA interaction may be involved in processes other than phagocytosis escape during *E. faecalis* infection. Together, our results pointed to ElrA acting as an anti-phagocytic cloak allowing *E. faecalis* to be undetected by host cells.

*E. faecalis* strains synthesizing a capsule have a reduced complement and antibody-mediated opsonization compared to unencapsulated strains^15,16^. Using genetically engineered immunocompromised zebrafish, the Epa rhamnopolysaccharide was also shown to allow host phagocytosis evasion^17^. In this work, we used the unencapsulated strain OG1RF and found that ElrA expression *in vitro* does not impact on the production of Enterococcal polysaccharide antigen (Epa) and of teichoïc acids present in *E. faecalis* envelope. The integrity of these latter components suggests that ElrA-expressing *E. faecalis* has the potential to remain phagocytized even if we cannot exclude that peptidoglycan-bound ElrA may mask PAMPs (Pathogen Associated Molecular Patterns) at the bacterial surface. In this study, we also found that at neutral pH ElrA expression does not alter the global charge, which remains negative and identical to that of bacteria not expressing ElrA. Previous physico-chemical studies have shown that *E. faecalis* has also a predominant negative charge at its surface^18,19^. It has been recently shown that anchored proteins in the host cell membrane provide a local positive charge indicating that negatively-charged pathogens can efficiently infect eukaryotic cells, despite a global negatively charged surface^20^.

To our knowledge, we report for the first time an inhibition of macrophage sensing triggered by an internalin-like protein. An inhibition of chemoattraction of macrophages towards bacteria has been observed in *S. aureus*, where the secretion of chemotaxis inhibitory protein (CHIP) and formyl peptide receptor-like 1 inhibitors bind to the formylated peptide receptor directly avoiding chemoattraction of neutrophils^21^. Moreover, the secretion of the cysteine protease staphopain cleaves CXCR2 chemokine reducing indirectly cell migration towards the infection site^22^. It has been previously demonstrated that the host cells can respond to *Enterococcus faecium* and *E. faecalis* infection through the activation of formyl peptide receptor-2^23^, where neutrophils are chemoattracted towards the infection site. In addition*, E. faecalis* has been shown to produce nonformylated peptides for neutrophil chemoattraction^24^. We found that ElrA affects *E. faecalis* capacity to form biofilms and diminishes the cohesion between bacteria. Biofilms are usually robust foci resisting host immune defenses^25^. However, planktonic *E. faecalis* seems less phagocytized when forming biofilm, indicating that the role of enterococcal biofilms in infection is not completely understood^26^. A hypothesis would be that poorly cohesive *E. faecalis* do not generate appropriate signals to trigger macrophage sensing and migration.

The role of ElrA in *E. faecalis* virulence is well documented and supports a role of ElrA in *E. faecalis* virulence^5,9,10^. Yet, the different mouse genetic backgrounds together with different routes of infection and different kinetics mimicking different stages of the immune response show different effects of ElrA disruption. Accordingly, we observed a minor role of ElrA in the liver of C57BL/6 mice infected by the intravenous route for 24 h. Role of FHL2, a eukaryotic partner of ElrA, in bacterial infection emerges^27^. We were able to show that FHL2 is implicated in host defense against *E. faecalis*. However, we observed a limited contribution of the interaction between ElrA and FHL2. FHL2 has been linked to pulmonary infection by *Schistosoma mansoni* where it affects macrophage polarization and modulates granuloma formation^28^. Here, we found that FHL2-deficient macrophages are still able to sense wild type *E. faecalis*. Non-covalent attachment of WxL proteins, like ElrA, results in the release of unbound proteins in the culture medium^7,29^. Since FHL2 is found below the plasma membrane, and in the cytosol and nucleus of the cell and ElrA is located at the bacterial surface or released in the culture medium, it is likely that FHL2-ElrA interaction occurs once *E. faecalis* has been internalized. We propose that ElrA may exert two distinct functions. When bacteria are outside the cells, ElrA acts as an anti-phagocytic cloak, which does not involve FHL2. The other unidentified function would be for internalized bacteria to enhance bacterial survival, a function that would be counteracted by FHL2. Together our results indicate at least a dual function of ElrA depending on the extracellular versus intracellular *E. faecalis* location during infection.

## Methods

### Bacterial strains

*E. faecalis* strains used in this study are listed in Table 1. *E*. *faecalis* was routinely grown in Brain Heart Infusion (BHI) medium at 37 °C without aeration. The P^+^-Δ*elrA* strain was complemented for ElrA expression by electroporation with pVE14132 (CPL-*elrA*)^5^ and selected with 10 µg/ml erythromycin. *Escherichia coli* was grown aerobically in Luria-Bertani (LB) medium at 37 °C. For heterologous ElrA expression, *E. coli* BL21(DE3) was transformed with the plasmid pVE14047 encoding 6XHis-Tag-ElrA deleted of its signal peptide and selected using 100 µg/ml ampicillin.

### Cell lines

The murine Raw 264.7 macrophage cell line was grown in Dulbecco’s Modified Eagle Medium (DMEM, Gibco) with 10% decomplemented fetal bovine serum (FBS, Sigma), 2 mM glutamine and 25 mM Hepes. The human ileocecal adenocarcinoma (HCT-8) cell line was grown in DMEM with 10% FBS and 2 mM glutamine. The human placenta choriocarcinoma (Jeg-3), kidney adenocarcinoma (A-704) and liver hepatocellular carcinoma (HepG2) cell lines were grown in Minimum Essential Medium (MEM, Gibco) supplemented with 10% FBS, 2 mM glutamine, 0.1 mM non essential amino acids and 1 mM sodium pyruvate. The human epithelial colorectal adenocarcinoma (Caco-2) cells were grown in MEM with 20% FBS, 2 mM glutamine, 0.1 mM non-essential amino acids and 1 mM sodium pyruvate. The human epitheloid cervix carcinoma (HeLa) and kidney adenocarcinoma (ACHN) cell lines were grown in MEM with 10% FBS, 2 mM glutamine and 0.1 mM non-essential amino acids. L929 cells were grown in DMEM supplemented with glutamine 1 mM, Penicillin and Streptomycin 100 U/ml, HEPES 25 mM and 5% FBS. After 10 days at confluence, cell supernatants containing the M-CSF for monocyte differentiation into macrophages were recovered and centrifuged at 750 g for 10 min. All cells were incubated at 37 °C in 5% CO_2_ atmosphere.

### Construction of the pVE14047 plasmid

A DNA fragment containing the *elrA* open reading frame minus the predicted signal peptide was amplified by PCR from *E. faecalis* OG1RF genomic DNA using primers OEF275 (5′-CAAACATGTTAGAAACGACCGAAACAATCGC) and OEF276 (5′-TTGGATCCACTCACCCCCTATTTTGC). The resulting PCR fragment was digested with *AflIII* and *BamHI* and inserted into the pET2817 vector to generate pVE14047, which encodes the 6xHistidine-tagged-ElrA. After verification by sequencing, pVE14047 was introduced into *E. coli* BL21 (DE3) and transformants were selected using ampicillin 100 µg/ml.

### Expression and purification of ElrA and rabbit anti-ElrA antibodies

Bacterial culture (1 L) of pVE14047-containing *E. coli* was grown at 23 °C to OD_600_ = 0.6. Overexpression of the 6xHis-Tag-ElrA was induced by addition of 1 mM Isopropyl β-D-1-thiogalactopyranoside (IPTG). Bacteria were harvested at OD_600_ = 1 by centrifugation at 4000 g for 20 min at 4 °C and resuspended in 17 ml phosphate buffer containing 5 mM imidazole, 10 µg/ml RNAse A (Sigma), 5 µg/ml DNase I (Roche), Halt Protease and Phosphatase inhibitor (Thermo Scientific). Bacteria were disrupted by sonication (20% amplitude) with the Vibro cell 75042 (Bioblock Scientific). Lysates were recovered after centrifugation at 8600 g for 20 min at 4 °C and loaded for 1.5 h on 4 ml of cobalt affinity resin (Ozyme) in 5 mM imidazole. The column was then washed 4 times with a solution of 10 mM imidazole, 300 mM NaCl and 50 mM NaH_2_PO_4_-H_2_O, pH 8, four times in 20 mM imidazole, 300 mM NaCl and 50 mM NaH_2_PO_4_-H_2_O, and twice in 20 mM imidazole, 1 M NaCl, 50 mM NaH_2_PO_4_-H_2_O. ElrA was then eluted with a gradient of imidazole from 50 to 300 mM in 300 mM NaCl and 50 mM NaH_2_PO_4_-H_2_O solution. Purified fractions were dialysed using a Pierce Slide-A-Lysed cassette in 50 mM Tris-HCl, 150 mM NaCl pH 7.5 overnight at 4 °C. Protein was concentrated in Amicon Ultra-15 Centrifugal Filter devices at 4000 g for 20 min at 4 °C. This method allowed us to purify approximately 4 mg of recombinant ElrA from 1 L of culture. Rabbit anti-ElrA antibodies have been developed by Covalab SAS.

### Protein extraction and Western blot

All strains were recovered from exponential growth phase (OD_600_ = 0.8) by centrifugation at 8600 g for 20 min at 4 °C. Bacterial pellets (1 ml) were diluted in 500 μl of Laemmli buffer containing 0.25 M dithiothreitol (Sigma) and protease inhibitors (Halt cocktail 1×) and kept on ice. Samples were transferred to 2-ml screw-cap micro centrifuge containing silica beads (Lysing Matrix B tubes, MP Biomedicals). Bacteria were disrupted for 45 sec at level 6 with the Fast Prep 24 apparatus (MP Biomedicals). All tubes were then centrifuged at 13500 rpm for 10 min. Soluble protein extracts (50 μl) were separated on a 12% polyacrylamide gel and transferred on a cellulose membrane for 12 min at 25 V and 1.6 A using Pierce G2 Fast Blotter (Thermo Scientific). Membranes were blocked 8 hours using 5% milk in PBS-Tween 0.1%. ElrA was revealed overnight at 4 °C using the enriched fraction of anti-ElrA antibodies (1/100), precleared for 3 h at 4 °C in 500 μl of total protein extract of the ΔElrA strain in PBS tween 0.01%. The day after, we used a secondary goat anti-rabbit antibody conjugated with horseradish peroxidase (#BI 2411, Abliance) at 1:5000 in 5% milk in PBS-Tween 0.1%. Membranes were revealed using Immobilon Western Chemiluminescent HRP Substrates (Millipore) with a ChemiDoc MP system (Bio-Rad).

### Zeta potential

Zeta potential of the different *E. faecalis* strains was measured from bacteria harvested by centrifugation at OD_600_ = 0.6 as previously described^30^. Briefly, pellets were washed twice and resuspended in DMEM 1:50 in MilliQ water (Millipore™) and were submitted to an electric voltage (50 V). Electrophoretic motility was measured using a zetaphoremeter (Cad Instruments, Les Essarts le Roy) at pH 7. Mean values and SEM were obtained from two independent experiments.

### Polysaccharide analysis

Bacteria were harvested 1 h after the stationary phase, washed and resuspended in 5 mg/ml lysozyme (Sigma) and 250 U/ml mutanolysin (Sigma) in PBS and incubated at 37 °C overnight. Next, polysaccharides were extracted and revealed as previously described^31^.

### Electron microscopy

Bacterial pellets harvested at OD_600_ = 0.6 were washed with phosphate-buffered saline (PBS) solution and fixed with 2% glutaraldehyde in 0.1 M Na cacodylate buffer pH 7.2, for 1 h at room temperature. Samples were treated as previously described^32^. Thin sections (70 nm) were collected onto 200 mesh copper grids, and counterstained with lead citrate. Grids were examined with Hitachi HT7700 electron microscope operated at 80 kV (Elexience – France), and images were acquired with a charge-coupled device camera (AMT).

### Biofilm formation and analysis

Biofilm formation has been investigated as previously described^33^. Briefly, stationary phase *E. faecalis* cultures were used to freshly inoculate BHI in 96-well microplates (1:100). Bacteria attached to the wells (in triplicate) for 1 h at 37 °C and were washed once with fresh medium. Biofilms developed for 6 h were stained using SYTO 9 (cell permeant nucleic acids dye, Life Technologies) diluted 1:100. Images were acquired using a Leica SP8 confocal laser scanning microscope (Leica Microsystems). The green SYTO 9 emitted fluorescence signal was collected on an hybrid detector in the range 500–550 nm after excitation at 488 nm with an Argon laser set at 20% of its maximal intensity with a z-step of 1 µm and at 600 Hz. 3D reconstructed images were obtained using Imaris (Bitplane) software. Biomass was calculated directly from the raw images using Image J software and Comstat 2 plugin^34^. Percentage of cohesion was calculated by normalizing the remaining biofilm after the three consecutive washes to the initial non-washed biofilm. Statistical tests were performed using unpaired Student’s t test on GraphPad Prism.

### Cell invasion assay

Two to 5 days before infection, cells were seeded in triplicate in 24-well plates. Raw267.4 macrophages were activated with 1 µg/ml of purified *E. coli* lipopolysaccharide (Sigma) for 24 h before bacterial contact. Prior to infection, all cells except macrophages were washed once with PBS and incubated in Krebs-Ringer buffer (Sigma) as described previously^35^. *E. faecalis* strains were grown to an OD_600_ of 0.6, washed twice in PBS and resuspended in medium without serum and used at a multiplicity of infection (MOI) of 5 for macrophages and 50 for epithelial and fibroblastic cells. After 1 min centrifugation at 1000 g and 1 h of contact, cells were washed 4 times with PBS and extracellular bacteria were killed using 150 µg/ml gentamycin and 10 µg/ml vancomycin antibiotic cocktail. Cells were lysed 1 hour post-infection with 0.5% Triton X-100 and intracellular bacteria enumerated on BHI agar plates. Percentage of invasion was determined as the ratio of intracellular bacteria to the initial inoculum. Statistic tests were performed using unpaired Student’s t test on GraphPad Prism.

### Bead phagocytosis assay

Raw264.7 macrophages were incubated for 3 hours with the P^+^-*elrA-E* strain (MOI 5) as described above or with purified ElrA (10 nM). Next, 0.1 µm fluorescent latex beads (Sigma) were added to an MOI of 5. To synchronize bead phagocytosis, plates were centrifuged 10 min at 400 g. One hour after contact, cells were washed four times with PBS and resuspended in PBS EDTA 2 mM. The percentage of bead-positive cells was determined using a FACSCalibur (BD Biosciences) after gating on live macrophages. Analysis was performed with FlowJo (Tree Star) software.

### Macrophage migration

To quantify macrophage migration towards bacteria, we performed a modified Boyden chamber assay^36^. Briefly, Raw264.7 macrophages, or bone marrow derived macrophage (BMDM) generated as previously described^37^, were adapted in 1% FBS containing medium for 6 h and recovered in DMEM without serum. 5 × 10^4^ cells were seeded on the upper chamber of a 24-well insert containing a 4 µm polyethylene terephthalate (PET) membrane (Millipore). The *elrA* isogenic strains (P^+^-*elrA-E*, P^+^-Δ*elrA* and CPL-*elrA*) were seeded in the bottom chamber at 5 × 10^5^ CFU (eq. to MOI of 10). After ~20 h of incubation, the medium of the upper chamber was plated to verify absence of bacteria. Then, wells were washed twice on PBS and fixed with 3.7% PFA for 2 min. Cells were permeabilised with 100% methanol for 20 min and stained with Giemsa stain for at least 2 h. Migrating cells were trapped in the membrane whereas non-migrating cells were scraped from the upper chamber using a cotton swab. Wells were directly observed using an inverted light microscope. Statistic tests were performed using unpaired Student’s t test on GraphPad Prism.

### Animal studies

All mice were housed under specific pathogen-free conditions and handled in accordance with French and European directives. Animal experiments were authorized by the Ministry of Education of Higher Education and Research (APAFIS # 480, protocol number 20150415180048149_v1). All mice were on C57BL/6 J background. FHL2^−/−^ mice have been generated by Chu *et al*.^38^ and have been housed at the CEPR (INSERM U1100, Tours) and at the Pasteur Institute. C57BL6/J control mice were purchased at Charles River. Only conventional 9-to 12-week-old male C57BL6/J and FHL2^−/−^ mice were used for experiments. *E. faecalis* OG1RF and Δ*elrA* strains were collected by centrifugation 1 h after they had reached the stationary phase. Bacterial cells were washed twice with PBS 1× and stored at −80 °C. Mice were infected intravenously in the retro-orbital vein with 5 × 10^8^ CFUs. Serial dilutions of the inoculum were plated to control the number of *E. faecalis* inoculated in mice. The liver, the spleen and the kidneys were recovered 24 hours post-infection. Serial dilutions of organ homogenates were plated on Bile Esculin Agar (BEA) plates, a selective differential agar used to isolate *E. faecalis*. Statistical analyses were performed using Mann-Whitney test on GraphPad Prism.

## Electronic supplementary material

Supplementary Figures
