# Supplementary material for: Exploration of the role of the virulence factor ElrA during Enterococcus faecalis cell infection
Source: Sci Rep. 2018 Jan 29;8:1749. doi: 10.1038/s41598-018-20206-6 (PMC5788860; doi:10.1038/s41598-018-20206-6)
Supplement: Supplementary file 1 — Supplementary Figures [file 41598_2018_20206_MOESM1_ESM.pdf]

Exploration of the role of the virulence factor ElrA during *Enterococcus faecalis* cell infection

Natalia Nunez[1], Aurélie Derré-Bobillot [1], Stéphane Gaubert[1], Jean-Marie Herry[1], Julien Deschamps[1], Yu Wei[2#], Thomas Baranek[3.4], Mustapha Si-Tahar[3.4], Romain Briandet[1], Pascale Serror[1,*] and Cristel Archambaud[1,*]

[1]Micalis Institute, INRA, AgroParisTech, Université Paris-Saclay, 78350 Jouy en Josas, France

[2] Département de Virologie, Institut Pasteur, 28 rue du Dr. Roux, 75015 Paris, France

[3]INSERM, Centre d'Etude des Pathologies Respiratoires (CEPR), UMR 1100, Tours, France

[4] Université François Rabelais, Tours

[#] Present address: Unit of Hepatitis B Virus and Liver Disease, Institut Pasteur of Shanghai, Chinese Academy of Sciences, 320 Yueyang Road, 200031 Shanghai, China

*Corresponding authors: cristel.archambaud@inra.fr ; pascale.serror@inra.fr

Tel :+33134652083

Fax : +33134652065

SUPPLEMENTAL FIGURE LEGENDS

Supp. Figure 1. Antibodies directed against the recombinant ElrA were used to detect native ElrA (80 kDa) from total protein extracts of the OG1RF wild-type strain not expressing the *elrA-E* operon *in vitro*; the P^+-*elrA-E* strain, which constitutively expresses the operon; the P^+-Δ*elrA* strain constitutively expressing *elrB-E* but not *elrA*; and the CPL-*elrA* strain that corresponds to P^+-Δ*elrA* complemented for *elrA* expression. Purified ElrA (30 ng) was loaded as a positive control. Positions of the molecular mass markers are indicated on the left.

Supp. Figure 2. Biofilms of ElrA isogenic strains were observed 6 h post inoculation at 37°C and after three consecutive washes. Bacteria were stained and biofilms were observed by confocal microscopy. Images were constructed using IMARIS software and the biomasses were calculated from using Image J. The percentage of cohesion was calculated using as a reference the initial biomass at 6 h without washes and compared to the remaining biomass after washes. Mean and standard deviations on 3 independent experiments are shown. Statistical analysis was performed using unpaired Student's t test. Asterisks indicate a p-value considered statistically significant (**, $P < 0.01$).

Supplementary Figure 1

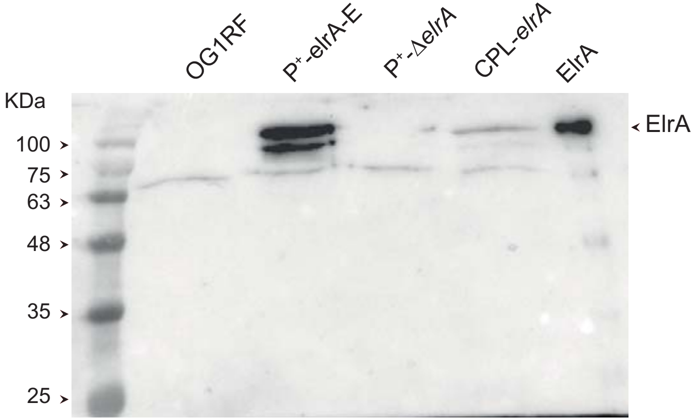

Supp. Figure 1. Antibodies directed against the recombinant ElrA were used to detect native ElrA (80 kDa) from total protein extracts of the OG1RF wild-type strain not expressing the *elrA-E* operon *in vitro*; the P$^+$-*elrA-E* strain, which constitutively expresses the operon; the P$^+$-Δ*elrA* strain constitutively expressing *elrB-E* but not *elrA*; and the CPL-*elrA* strain that corresponds to P$^+$-Δ*elrA* complemented for *elrA* expression. Purified ElrA (30 ng) was loaded as a positive control. Positions of the molecular mass markers are indicated on the left.

Supplementary Figure 2

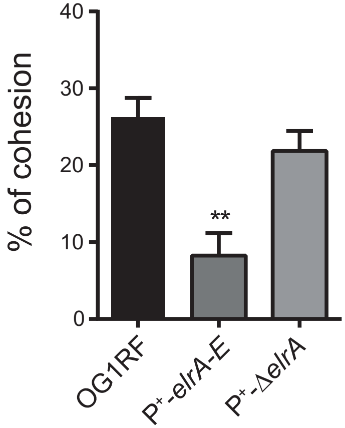

Supp. Figure 2. Biofilms of ElrA isogenic strains were observed 6 h post inoculation at 37°C and after three consecutive washes. Bacteria were stained and biofilms were observed by confocal microscopy. Images were constructed using IMARIS software and the biomasses were calculated from using Image J. The percentage of cohesion was calculated using as a reference the initial biomass at 6 h without washes and compared to the remaining biomass after washes. Mean and standard deviations on 3 independent experiments are shown. Statistical analysis was performed using unpaired Student's t test. Asterisks indicate a p-value considered statistically significant (**, $P < 0.01$).
